# Research into Preparation and Performance of Fast-Hardening RPC Mixed with Straw

**DOI:** 10.3390/ma16155310

**Published:** 2023-07-28

**Authors:** Kaiwei Gong, Zhao Liang, Xi Peng, Hui Wang

**Affiliations:** 1 School of Civil Engineering and Geographical Environment, Ningbo University, Ningbo 315000, China; 1095957844@aliyun.com (K.G.); liangzhao9915@aliyun.com (Z.L.); 2 School of Civil Transportation Engineering, Ningbo University of Technology, Ningbo 315211, China; 3 Engineering Research Center of Industrial Construction in Civil Engineering of Zhejiang, Ningbo University of Technology, Ningbo 315048, China

**Keywords:** fast-hardening RPC mixed with straw, working performance, mechanical performance, compactness and hydration products

## Abstract

Based on its characteristics of early strength, good toughness, and excellent mechanical and impact resistance, steel fiber-reinforced fast-hardening reactive powder concrete (RPC) is expected to become an alternative material used in the rapid repair of marine concrete structures. However, the steel fibers have also caused corrosion problems in coastal environments. To make doped fiber fast-hardening RPC more adaptable for use in ocean engineering, this study prepares fast-hardening RPC mixed with straw and studied the effects of straw content and curing age on its slump flow, setting time, and mechanical performance (flexural strength, compressive strength, and flexural toughness). The effects of straw addition on the compactness and hydration products of fast-hardening RPC were studied through macro- (ultrasonic analysis) and micro-scopic analysis (electron microscopy scanning and X-ray diffraction patterns). The straw content mentioned in this paper refers to the percentage of straw in relation to RPC volume. The results showed that straw reduced the fluidity of RPC slurry by 10.5–11.5% compared to concrete without straw, and it accelerated the initial setting of RPC slurry. When the straw content accounted for 1% of RPC volume, the setting rate was the fastest, with a increasing rate being 6–18%. Compared to concrete without straw, the flexural and compressive strength of fast-hardening RPC was enhanced by 3.7–30.5%. When the content was either 3% or 4%, the mechanical properties improved. Moreover, when the straw content accounted for 4% of RPC volume, the flexural toughness was the highest, with the increase rate being 21.4% compared to concrete without straw. Straw reduces the compactness of fast-hardening RPC.

## 1. Introduction

Since the country’s reform and opening up process began, China’s infrastructure construction has rapidly developed, and many large concrete buildings have been built in coastal areas, such as the Hangzhou Bay Bridge, Ningbo; the Hong Kong–Zhuhai–Macao Bridge; and the Jiaozhou Bay Bridge, Qingdao. Such buildings often have the characteristics of being the result of huge financial investment, complex structures, long design lives, etc., which suggests that they must have high durability and long service lives. However, during long-term service, the durability of such buildings is often significantly affected by the erosion of salt in seawater and the existence of cyclic loads such as wind load, current impact [1], and seismic wave [2]. In addition, the northern offshore structures are often affected by freeze-thaw cycles and have a certain coupling effect related to loads, which further aggravate the damage of such structures. In short, the existence of these problems has significantly affected the service lives of offshore concrete structures. In order to realize the rapid repair of bridges and other buildings and structures without affecting normal traffic flow and use, steel fiber fast-hardening active powder concrete is often used as the repair material. However, engineering practice has found that steel fiber RPC has serious corrosion problems in marine environments [3,4,5], which has forced many scholars to reconsider its applicability in marine environments and find a new type of seawater erosion-resistant fiber materials.

Research into the incorporation of fiber into concrete has also been widely conducted by researchers in countries around the world, including the United States, Japan, and the United Kingdom [6,7,8], and these studies can provide references to help us to find new fiber materials resistant to seawater erosion. For example, Wu found that adding fibers to basalt fiber-reinforced concrete significantly improved the toughness and crack resistance of the concrete, with the optimal fiber content being 0.25% and the maximum increase in compressive strength being 4.48% [9]. In addition, a study has shown that adding polyester fibers to concrete during preparations, which increased the tensile strength and low-temperature bearing capacity by 10.3% and 55.3%, respectively [10]. Moreover, studies of modified carbon nanotube fiber-reinforced polymer (MCNF) also found that its dynamic compressive strength was 14.0–35.5% higher than that of untreated concrete [11]. Compared to ordinary concrete, steel fiber concrete has been greatly improved in terms of its physical and mechanical properties, the acquisition of steel fiber is relatively simple, and the cost of preparation is not high. Thus, the use of steel fiber concrete is more common than that of ordinary concrete in some areas that have high performance requirements. However, due to long-term seawater immersion, dry and wet alternating, and the freeze–thaw cycle, the addition of steel fiber will also affect the durability of concrete in marine environments [12,13,14].

At present, there are relatively few studies of straw fiber concrete. Straw, which is a type of waste after crop harvesting, has good heat insulation and short setting time compared to steel fibers and some polymer fibers [15]. However, its improper treatment can often cause environmental pollution [16,17]. Muhammad found that the compressive and flexural strength and crack toughness indices increased by 91%, 92%, and 105% with 1% straw content. [18]. Liu found that compared to ordinary straw fiber concrete, the 28-day compressive strength of 3% nano-silica concrete that contained 2% straw fiber content increased by 28.6%, and the flexural strength increased by 27.9% [19]. The rapeseed straw fiber concrete studied by Hajj had low thermal conductivity, with an average of 0.073 W·m^−1^·K^−1^ [20]. In addition, it has been reported that experiments using NaOH to treat straw concrete have achieved higher density, splitting strength, tensile strength, and compressive strength [21]. It can be seen that straw fiber concrete has attracted the attention of most scholars and has relatively good performance. In order to respond to the theme of “carbon neutrality and carbon peak” in China, the research into straw fiber needs to be more in- depth in nature.

The addition of straw fiber can improve concrete’s performance, though it is still difficult to make ordinary concrete meet the needs of marine environments. Reactive powder concrete is a new type of cement-based composite material that has many excellent properties, such as high strength, high toughness, high durability, and good volume stability. Thus far, there has been some systematic research into its strength, toughness, durability, and other characteristics [22,23,24,25,26], which can meet the high- strength requirements of the marine environment. However, there are currently few reports on the preparation of straw-based fast-strength reactive powder concrete by combining rapid hardening reactive powder concrete with straw fiber concrete.

In the marine environment, the early strength of concrete is conducive to construction. However, the reactive powder concrete does not have early strength properties. At present, the preparation of common fast-hardening concrete is mainly divided into three categories. For example, the freeze-resistant fast-hardening concrete developed by Abdraimov Company has the characteristics of fast hardening and frost resistance [27], which is achieved by using fast-hardening and early strength special cement. Secondly, fast-hardening concrete can also be prepared using early strength agents, including nitrite, chloride, silicate, sulfate, and other inorganic salt early strength agents [28]. Research has shown that composite early strength agents can significantly improve their early strength performance. Finally, fast-hardening concrete can also be achieved through special curing methods. Tang found that ultra early strength cement concrete obtained by spraying the curing agent once after pouring can achieve the best curing effect [29]. Researchers have also found that the higher the curing temperature, the greater the strength of concrete [30]. Using the three above-mentioned strategies, the straw-doped reactive powder concrete can be made to have early strength performance to allow it to adapt to marine environments. This paper adopts the strategy of adding an early strength agent.

This experiment measured the mechanical performance (flexural and compressive strengths and flexural toughness) of fast-hardening reactive powder concrete with straw contents of 0%, 1%, 2%, 3%, and 4%. The compactness of fast-hardening RPC under different straw contents was analyzed from both macro- and micro-based perspectives through ultrasound and electron microscopy scanning experiments. In addition, the hydration products of fast-hardening RPC when using different straw contents were studied through X-ray diffraction patterns experiments. The content of this paper outlines a possible way to select the best straw fiber content to quickly repair marine structures in the future.

## 2. Experimental

### 2.1. Raw Materials

Conch-brand ordinary silicate cement with a strength grade of P42.5 and silica fume was used as the cementitious material. The characteristics of raw materials used are shown in Table 1. Aggregate is the quartz sand produced by Ling Shou County Yongshun Fruit Products Processing Co., Ltd., Lingshou, China, and it has high activity [31]. The water-reducing, defoamer, and calcium formate early strength agents were produced by Shanghai Ying Shirt Material Co., Ltd., Shanghai, China. The cumulative passing rate and chemical composition of gelling materials are shown in Table 2 and Table 3.

### 2.2. Specimen Preparation

Reactive powder concrete was used as a cement based material by adding 0.53% of early strength agent, and, according to the volume ratio, these samples were mixed with 0%, 1%, 2%, 3%, or 4% straw fiber content preparation of proportion of five groups of straw fiber speed can reactive powder concrete samples, respectively for 1–5 groups. The main chemical composition of straw fiber was mainly made up of cellulose, hemifibrin, and lignin. The straw fiber was prepared in groups with volume percentages of 0–4%. The proportion of 1 m^3^ specimens used in each group is shown in Table 4, and the sand mass ratio in the table is shown as coarse:medium:fine = 1:1.5:0.8.

The weighed materials included gelling materials (ordinary Portland cement and silica fume), and quartz sand used in the experiments of each group were poured into the wet UJZ-15 vertical mortar mixer. Next, the water reducer, defoamer, and early strength agents were added into the water and mixed evenly to create a solution. Finally, the straw was sequentially added and stirred into the mixture for 480 s.

**Figure 1:**
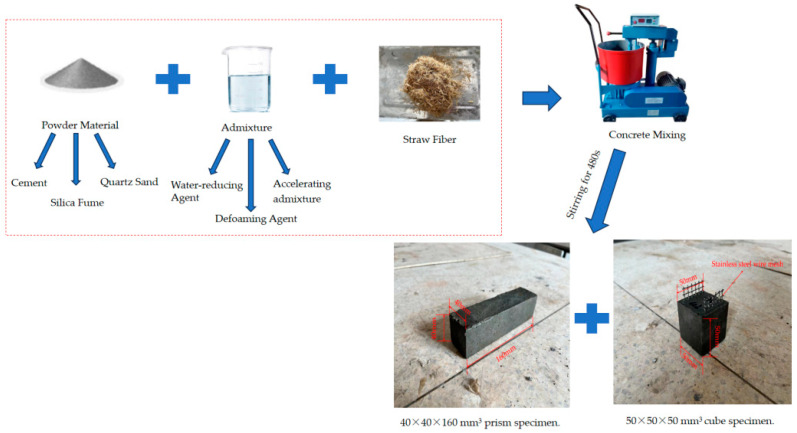
The process of producing fast-hardening RPC mixed with straw.

After the materials were stirred, the remaining fast-hardening RPC samples were poured into the oiled plastic molds, which were prism specimens of 40 mm × 40 mm × 160 mm and cube specimens of 50 mm × 50 mm × 50 mm, which were used for the flexural and compressive strength, flexural toughness, and ultrasonic measurement tests. All specimens were cured at a temperature of 20 ± 2 °C and relative humidity of over 95% after demolding. The whole production process is shown in Figure 1.

### 2.3. Measurement Method

**Figure 2:**
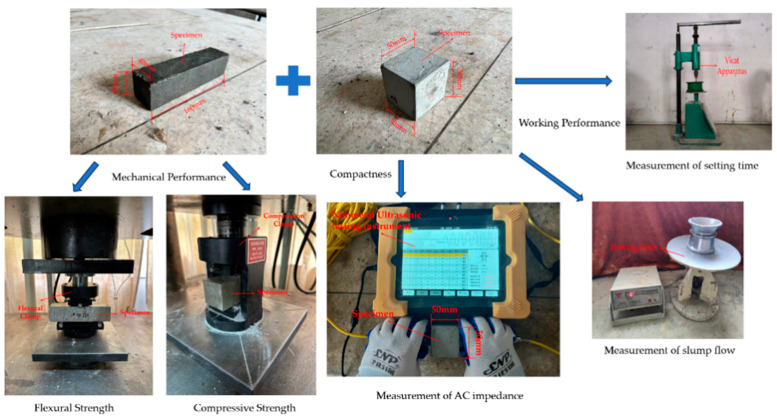
Experimental equipment used to determine slump flows, setting times, and mechanical properties.

The experimental equipment used are shown in Figure 2.

#### 2.3.1. Slump Flow and Setting Time Experiments

Even though RPC was concrete, after the mixer automatically stopped, the RPC slump flows of the newly mixed straw samples were measured according to GB/T2419-2005, i.e., “Method for Determining the Fluidity of Cement Mortar” [32].

The initial setting time of the newly mixed straw fast-strength RPC was determined according to JGJ70-90, i.e., “Basic Performance Test Method for Building Mortar” [33].

#### 2.3.2. Mechanical Property Experiments

**Figure 3:**
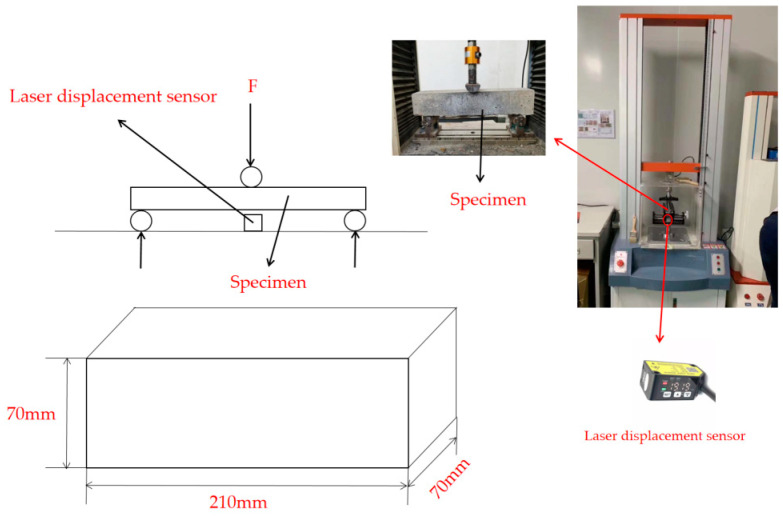
Three points bending test.

The compressive and flexural strengths of the specimens were tested using a WAW-600C microcomputer-controlled electro-hydraulic servo universal testing machine produced by Jinan Shijin Group Co., Ltd. Jinan, China. The dimensions of the fast-hardening RPC specimen were 40 mm × 40 mm × 160 mm. The concrete operation process was implemented with reference to the Chinese standard GB/T 17671-1999. The flexural toughness value of each group of samples was measured by integrating the curve segment located at the beginning of the bending force displacement curve, which was derived during the determination of flexural strength based on the highest point of loading. In addition, a three-point bending test was used to measure the bending toughness of each group of samples, and laser displacement sensor was used to measure the displacement. Three-point bending tests are shown in Figure 3.

#### 2.3.3. Compactness Experiments

The compactness of concrete was an important indicator used to evaluate the concrete. It was not only closely related to the mechanical properties of concrete [34], but also affected the durability of concrete [35,36]. In this experiment, the ZBL-U5100 non-metal Ultrasonic testing instrument produced by Beijing Zhibo Lian Technology Co., Ltd., Beijing, China, was used to conduct the ultrasonic velocity measurement experiment, electron microscope scanning, and the X-ray diffraction analysis experiment. The specific experimental process was previously referred to by Wang [37].

## 3. Results and Discussion

### 3.1. Slump Flow and Setting Time Analysis

**Figure 4:**
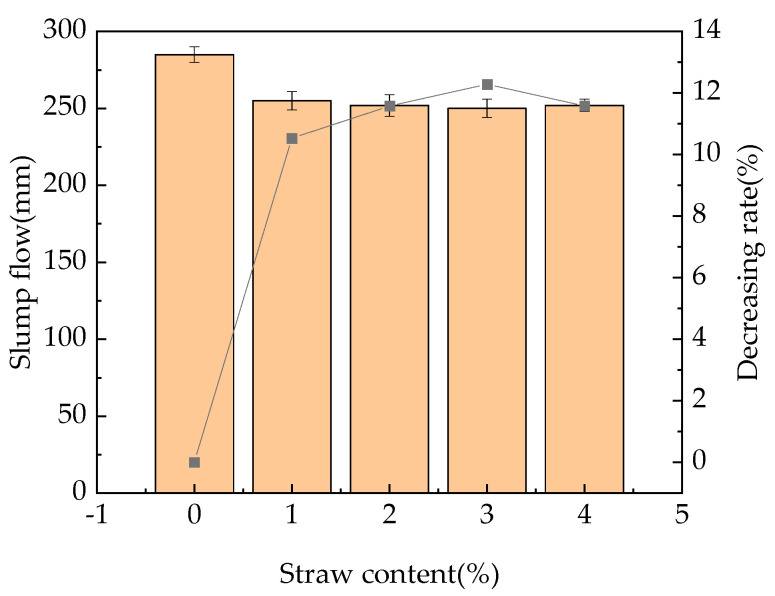
Relationship between the straw content and the RPC slump flow.

Figure 4 shows relationship between the straw content and the RPC slump flow.

It can be found that after the addition of straw fibers, except for straw content of 4%, the slump flow of fast-hardening RPC will decrease with the increase in straw content, with the decreasing rate being 10.5–11.5%. This result occurs because after the straw fiber is mixed into the mixture, when the original network-like C−S−H gel is formed, the straw fiber can bridge the fast-hardening RPC to form a spatial network-like structure, and there is a mechanical bite between the straw and the matrix, which leads to an increase in the internal friction of fast-hardening RPC and a reduction in its slump flow. Secondly, under the condition of a constant water–cement ratio in the experiment, as straw absorbs some water, the water absorbed by the cementitious material decreases, which reduces the fluidity of fast-hardening RPC, thus reducing its expansion. In addition, the slight fluctuation in the slump flow at a dose of 4% may be caused by the interaction between straw and silica fume.

**Figure 5:**
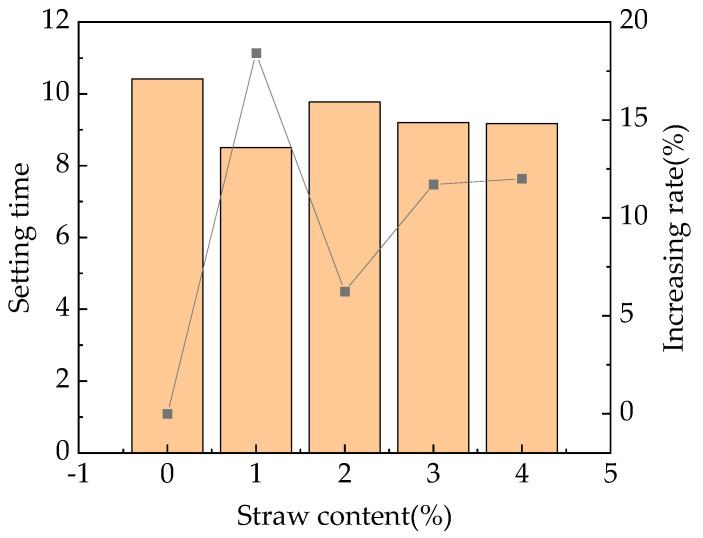
Relationship between the straw content and the RPC setting time.

In addition, as shown in Figure 5, the addition of straw fibers has a certain promoting effect on the initial setting of fast-hardening RPC, and with the increase in straw content, the setting time shows a trend of first decreasing, then increasing, and, finally, decreasing and tending toward a horizontal level, with the increasing rate being 6–18%.

The reason for this phenomenon is that when the straw content is less than 1%, due to the small straw content, the straw water absorption is lower, and the obstruction effect on the initial coagulation of the fast-hardening RPC is weak. At this time, the straw fiber has formed a relatively perfect spatial network-like structure in the fast-hardening RPC and has a large mechanical bite and friction force, thus significantly promoting the initial coagulation rate of the fast-strength RPC. However, when the straw content is between 1 and 2%, the straw fiber absorbs more water, the hindering effect on the initial coagulation of fast-hardening RPC is enhanced, the coagulation of fast-hardening RPC slows down, and the initial coagulation time is increased. When the straw content is more than 2%, because the spatial network structure-like formed by straw fiber in the fast-strength RPC is further improved, the mechanical occlusion and friction are further enhanced, the condensation is accelerated, and the initial setting time is shortened.

### 3.2. Mechanical Performance Analysis

**Figure 6:**
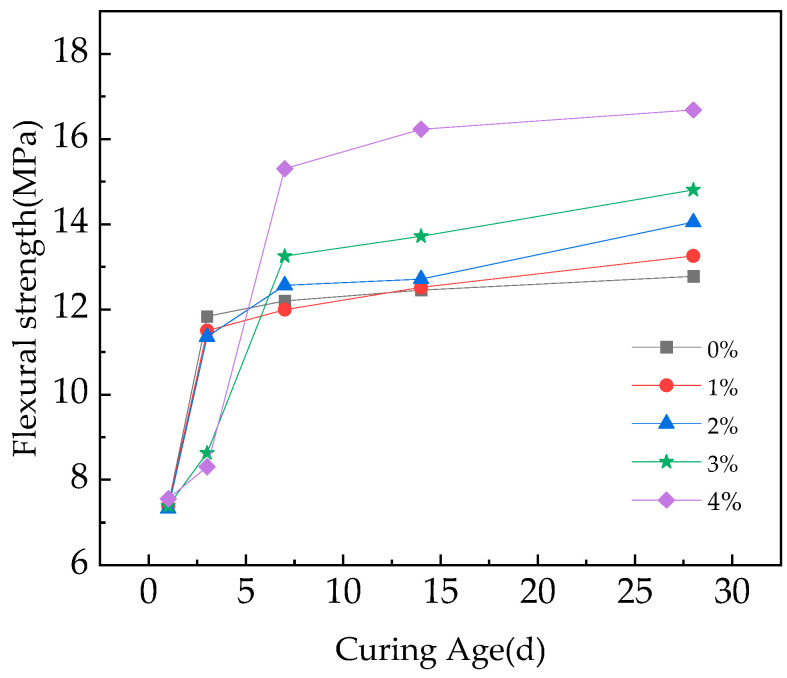
Flexural strength of RPC reinforced with different straw contents at different curing ages.

**Figure 7:**
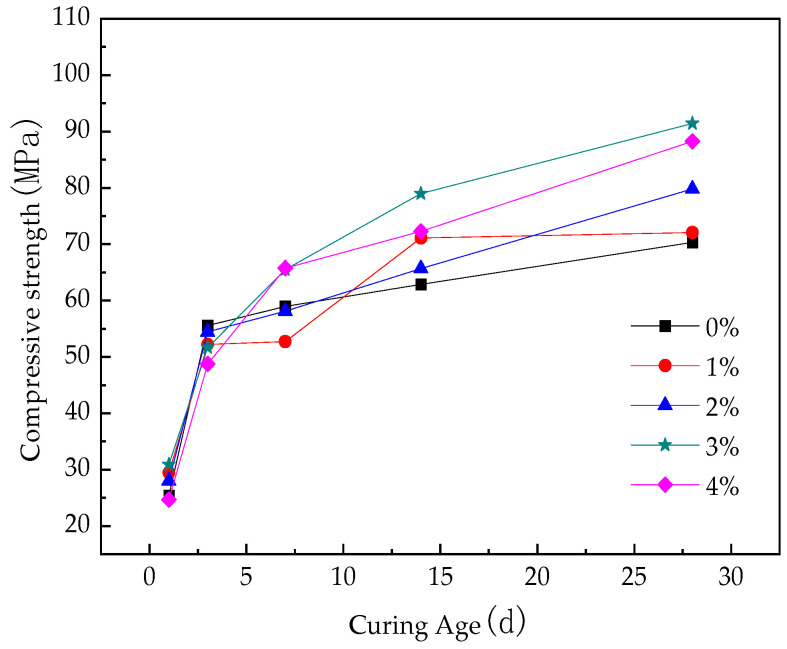
Compressive strength of RPC reinforced with different straw contents at different curing ages.

Figure 6 and Figure 7 show the flexural and compressive strengths with different straw contents at different curing ages.

Figure 6 and Figure 7 show the flexural and compressive strengths of RPC reinforced with different straw content at different curing ages. It can be seen that the straw content and curing age have the same significant influence on the flexural and compressive strengths of the fast-hardening RPC. Under the same straw content, with the increase in curing age, the strength of fast-hardening RPC increases at first and then tends to be stable. The reasons for this phenomenon are as follows: In the early stage, the strength of the fast-hardening RPC gradually increased in tandem with the condensation and hardening, the adhesion between the straw fiber and the fast-hardening RPC matrix increased, and the reinforcement effect increased, resulting in the strength gradually increasing. In the later stage, the hydration condensation of the fast-hardening RPC tended to complete, and the strength tended to remain unchanged.

In addition, at the same curing age, the flexural and compressive strengths of the fast-hardening RPC change based on the straw content. The overall trend is that the strength in the early stage decreases with the increase in straw content, and the strength in the later stage increases in tandem with the straw content, with the rate increasing by between 3.7 and 30.5%. The causes of this phenomenon can be considered to be based on two aspects: Firstly, straw water absorption hinders the condensation of fast-hardening RPC, which reduces its strength. Secondly, the stiffening effect of straw fiber in fast-hardening RPC improves its strength. In the early stage, due to the strong water absorption of straw, the hydration condensation of fast-hardening RPC was hindered, while the experimental water–cement ratio was unchanged. At this time, the bonding force between straw and fast-hardening RPC matrix was low, and the reinforcement effect of straw was weak. Therefore, the strength of fast-hardening RPC decreased with the increase in straw content at the same age. In the later stage, as the hydration condensation of the fast-hardening RPC tended to be completed, the bonding force between the straw and the fast-hardening RPC matrix was large and the stiffening effect was obvious, meaning that the bending strength of the straw increased with the increase in the straw content at the same age. It can be seen from the figure that when the straw content was either 1% or 2%, the influence of straw incorporation on the RPC strength was small. When the content of straw was either 3% or 4%, the influence of straw incorporation on the strength of RPC was greater. The flexural strength of RPC was largest in the later stage when the straw content was 4%, and the compressive strength was largest in the later stage when the content of straw was 3%.

### 3.3. Flexural Toughness Analysis

**Figure 8:**
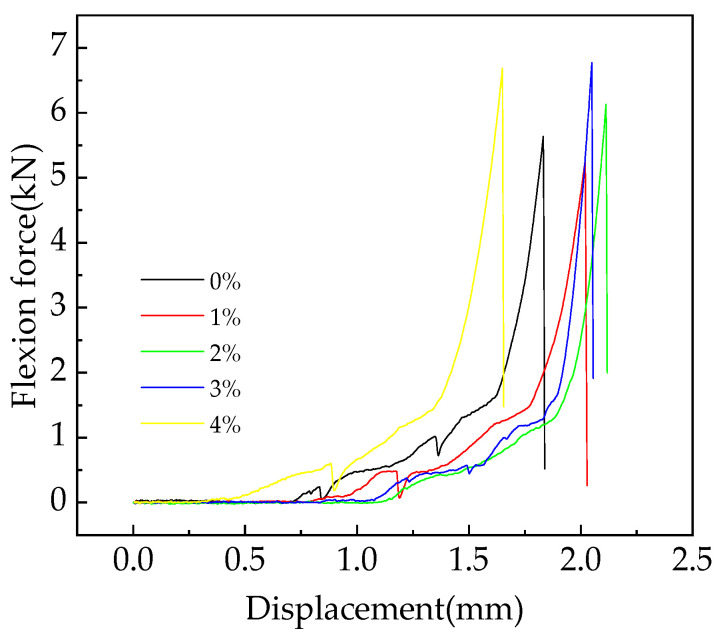
Relationship between flexion force and displacement for different straw contents.

Figure 8 shows the relationship between flexion force and displacement of fast-hardening RPC for different straw contents at a curing age of 28 days.

From the graph analysis, it can be seen that during the process of bending the specimen, the pressure exerted on the specimen shows a trend of being initially unchanged, before gradually increasing and, finally, sharply decreasing to zero with increasing displacement [38]. The reason for this phenomenon is that the experimental fixture did not contact the pressure surface of the universal testing machine in the early stage, and the pressure on the fast-hardening RPC specimen was zero. After contact, as the bending displacement increased, the force also gradually increased. When the specimen reached its maximum flexural strength, it suddenly broke, and the pressure on the specimen sharply dropped to zero.

**Figure 9:**
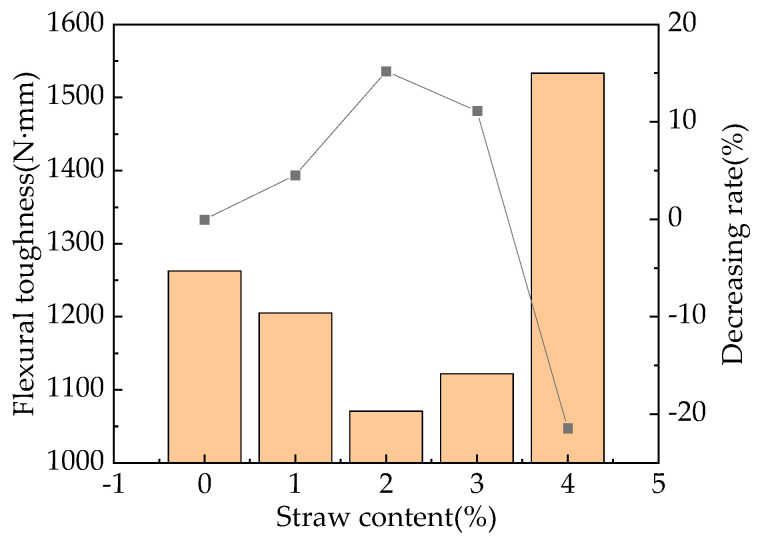
Relationship between the straw content and flexural toughness.

Without considering the influence of the tail of the curve, the flexural toughness values for RPC specimens with different straw contents that were cured for 28 days were calculated by integrating the bending force displacement line integral [39]. On this basis, the relationship between the straw content and flexural toughness of fast-hardening RPC cured for 28 days was determined, as shown in Figure 9.

Dong’s document can provide a detailed calculation method [40]. In addition, the measurement method of the three-point bending test can be learned from Wang [41]. The flexural toughness of fast-hardening RPC increases and then decreases with different doses of straw fiber. When the content of straw fiber is 2%, its flexural toughness is weakest, with the decreasing rate being 15.8%; in contrast, when the straw fiber content is 4%, the flexural toughness is greatest, with the increasing rate being 21.4%. The reason for this phenomenon is that due to the addition of straw, some air is introduced, resulting in a decrease in its density and strength, which weakens its bending toughness to some extent. The spatial network structure composed of straw fibers plays a certain reinforcing role, enhances its ductility, and, thus, enhances its bending toughness to some extent.

### 3.4. Microanalysis

**Figure 10:**
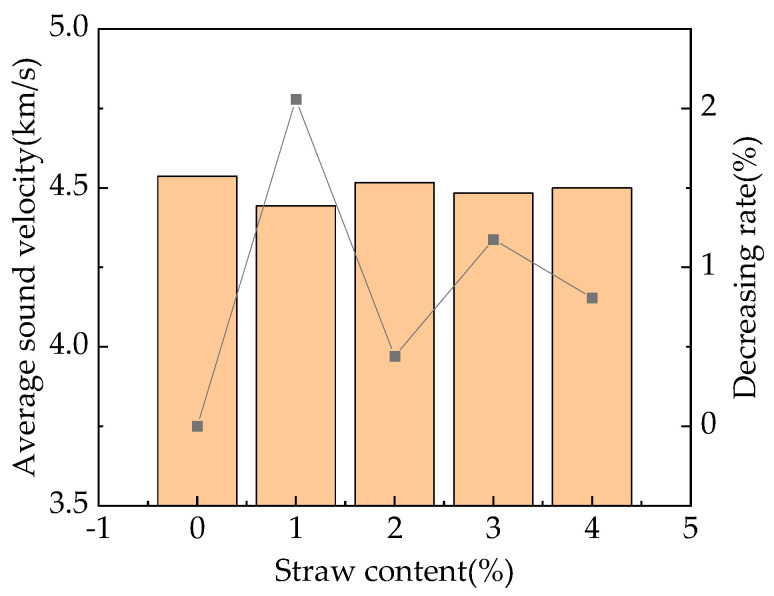
Relationship between the straw content and average sound velocity.

Figure 10 shows the average ultrasonic sound velocities of RPC specimens with a curing age of 28 days and straw contents of 0%, 1%, 2%, 3%, and 4%.

From Figure 10, it can be seen that except for the two groups of specimens with contents of 1% and 3%, which show increases in straw content, the overall RPC ultrasonic velocity shows a slow decreasing trend. This result was found because the introduction of air reduces the density and average sound velocity. Secondly, due to the absorption of some moisture by the straw and the organic matter of the straw itself having a certain stunting effect, the hydration of cement slows down, the density decreases, and the average sound velocity decreases, with the decreasing rate being 0.4–2.1%. Finally, due to the fact that the ultrasonic propagation rate of straw is lower than that of cement stone, after the addition of straw, some of the cement stone is replaced by straw, resulting in a decrease in the average ultrasonic propagation rate. This finding is consistent with the results of the compressive strength experiment of fast-strength RPC that we mentioned earlier in this study. After the addition of straw, the compressive strength of fast-strength RPC decreases. Moreover, the increase in flexural strength is due to the reinforcing effect of straw fibers within the RPC [42].

**Figure 11:**
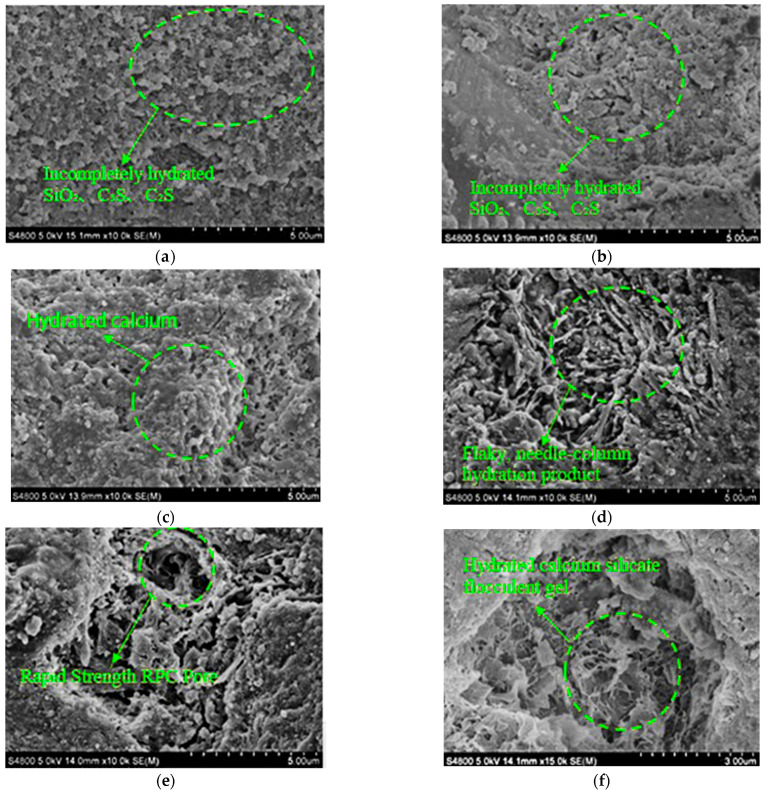
The SEM photo of RPC with different curing ages and straw contents. (**a**) Standard curing of 3 d− at 0%. (**b**) Standard curing of 3 d− at 2%. (**c**) Standard curing at 3d and −4%. (**d**) Standard curing at 28d− and 0%. (**e**) Standard curing at 28d− and 2%. (**f**) Standard curing at 28d− and 4%.

Figure 11 shows the SEM photos of RPC at curing ages of 3 days and 28 days and straw contents of 0%, 2%, and 4%.

As shown in Figure 11, when the curing period is the same (3 or 28 d), the compactness of fast-hardening RPC shows a gradually decreasing trend with the increase in straw content [43]. The reasons for this phenomenon can be attributed to three aspects. Firstly, due to the addition of straw, part of the air is introduced into the interior of the fast-hardening RPC, and the compactness is reduced. Secondly, when the experimental water–cement ratio is unchanged, due to the large water absorption of the straw, some of the water is absorbed by the straw, and the water used for cement hydration is reduced. In addition, the organic matter of the straw itself has a certain stunting effect, the hydration condensation of the fast-hardening RPC is hindered, and the compactness is reduced. It can be seen that the addition of straw reduces the compactness of the fast-strength RPC to a certain extent and affects its mechanical properties. This finding is consistent with the results of the above medium-speed strong RPC compressive test. After the addition of straw, the compressive strength of fast-hardening RPC decreases, and the increase in flexural strength occurs due to the reinforcement effect of straw fiber in its interior [44]. In addition, it can be seen from Figure 11 that when the curing period is 28 days, the fast-hardening RPC hydration condensation has approached completion. Compared to the fast-hardening RPC samples with a curing period of 3 days, more flake- and needle-shaped hydration product crystals were produced.

**Figure 12:**
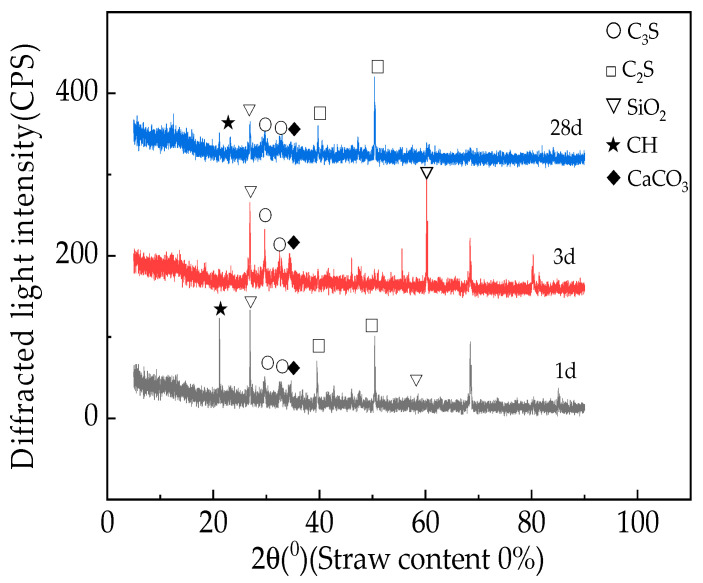
X-ray diffraction patterns of RPC with straw content of 0%.

**Figure 13:**
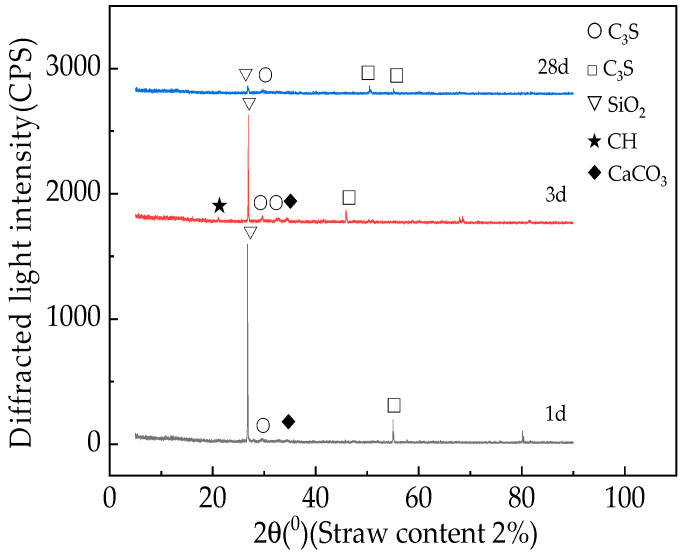
X-ray diffraction patterns of RPC with straw content of 2%.

Figure 12 and Figure 13 show the X-ray diffraction patterns of fast-hardening RPC with straw contents of 0% and 2% and curing periods of 1 day, 3 days, and 28 days [45].

Based on the analysis presented in Figure 13, it can be seen that when the straw content remains unchanged, the relative content of SiO_2_ in the fast-hardening RPC gradually decreases with the curing age. This process occurs because during the cement hydration and setting process, C_3_S and C_2_S hydrate to produce Ca(OH)_2_, while SiO_2_ reacts with Ca(OH)_2_ and gradually consumes it. As the fast-hardening RPC hydration condensation process progresses, the relative content of SiO_2_ gradually decreases [46].

**Figure 14:**
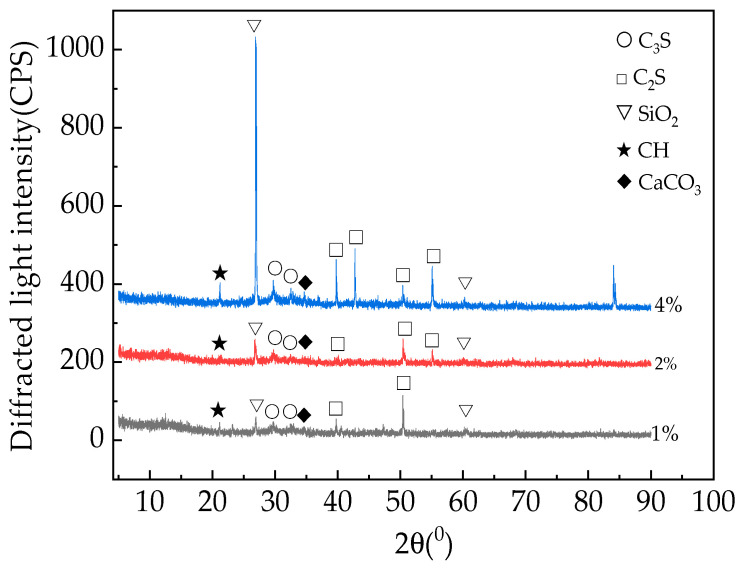
X-ray diffraction patterns of RPC with different straw contents.

Figure 14 shows the fast-hardening RPC X-ray diffraction patterns with a curing age of 28 days and straw contents of 0%, 2%, and 4%.

Based on the analysis presented in Figure 14, it can be seen that when the curing age of the fast strength RPC remains unchanged, the relative contents of C_3_S, C_2_S, SiO_2_, and Ca (OH)_2_ in the fast-hardening RPC sample show a gradually increasing trend in tandem with the increase in straw content. This process occurs due to the water absorption of straw fibers and the stunting effect of organic matter on the straw itself, which hinders the hydration and coagulation of fast-hardening RPC. At the same curing age, the consumption of C_3_S, C_2_S, SiO_2_, and Ca (OH)_2_ decreases.

## 4. Conclusions

In this experiment, the various properties of fast-hardening RPC mixed with straw are studied, and the results are as follows.

Straw fiber reduced the slump flow of newly mixed fast-hardening RPC, showing a decreasing rate of 10.5–11.5%, and the slump flow of fast-hardening RPC was smallest when the straw content was 3%. This mixture will reduce the hydration heat reaction of concrete and ensure the strength of the RPC. Straw fiber accelerates the mixing speed and strength of the RPC at the time of initial setting. When the straw content is 1%, the initial setting time of concrete is the shortest, and the increasing rate of the setting time is 6–18%. It will enable quick repair and ensure adequate strength of the concrete used in coastal environments.

The addition of straw has to some extent enhanced the flexural and compressive strength of fast-hardening RPC, with the rate increasing by 3.7–30.5%. When the straw content is either 3% or 4%, the flexural and compressive strength measures are most effective. In addition, the flexural toughness of RPC with straw content is closely related to the straw fiber content. Except for an increase of 21.4% in flexural toughness when the straw content is 4%, the flexural toughness of other straw content is weakened, and the flexural toughness is the worst when the straw fiber content is 2%. The content of straw should be considered based on the requirements of bending resistance and compressive strength.

The addition of straw reduces the compactness of fast-hardening RPC. Overall, the more straw is added, the more significant the decrease in compactness.

Straw fibers affect the hydration products of fast-hardening RPC. At the same curing age, the addition of straw increases the C_3_S, C_2_S, SiO_2_, and Ca(OH)_2_ contents in fast-hardening RPC.

## Figures and Tables

**Table 1:** The characteristics of raw materials.

Experimental Material	Characteristic
Cement	Specific surface: ≤350 m^2^/kg
Silica fume	Specific surface: 15 m^2^/g; density: 2.2 g/cm^3^; SiO_2_ content: ≥98%
Aggregate	Particle size: 1–0.71 mm, 0.59–0.35 mm, 0.15–0.297 mm; ratio: 1:1.5:0.8; main composition: SiO_2_ 99.66%, Fe_2_O_3_ 0.02%; specific surface: 2.5–3 m^2^/g; density: 2.2–2.3 g/cm^3^
Straw	Density: 35 kg/m^3^; breaking strength: 3.86 cN/dtex^−1^; length: 1–2 cm; bulk density: 0.2334 g/cm^3^
Water-reducing agent	High-efficiency water-reducing agent (SP) of polycarboxylic acid with a water reduction rate of up to 40% (teal micro-emulsion solution)
Defoamer	A mixture of polyether surfactant and dry powder carrier (white to light gray powder)

**Table 2:** Cumulative passing rate of the cementitious materials (/%).

	Particle Size/um	0.3	0.6	1	4	8	16	32	64	360
Category										
Silicate cement		0	0.33	2.66	15.01	28.77	46.64	72.73	93.59	100
Silica fume		31.2	58.3	82.3	100	100	100	100	100	100

**Table 3:** Chemical composition of the cementitious materials.

Category	Chemical Composition/%								
	SiO_2_	Al_2_O_3_	Fe_2_O_3_	MgO	CaO	SO_3_	R_2_O	MnO	H_2_O
Silicate cement	20.89	5.44	3.96	62.24	1.71	2.65	0.48	0	0
Silica fume	90	0.8	0.6	0.8	0.4	0	7.4	0	0

**Table 4:** Mixing proportion of straw-doped fast-hardening RPC/(kg/m^3^).

Group	Water	Cement	Silica Fume	Quartz Sand	Water Reducer	Early Strength Agent	Defoamer	Straw
1	1.500	4.500	1.500	3.000	0.060	0.0318	0.006	0
2	1.500	4.500	1.500	3.000	0.060	0.0318	0.00	0.0021
3	1.500	4.500	1.500	3.000	0.060	0.0318	0.006	0.0042
4	1.500	4.500	1.500	3.000	0.060	0.0318	0.006	0.0062
5	1.500	4.500	1.500	3.000	0.060	0.0318	0.006	0.0084

## Data Availability

The data used to support the findings of this study are available on request.
